# Comparison of ^18^F-FDG PET and arterial spin labeling MRI in evaluating Alzheimer’s disease and amnestic mild cognitive impairment using integrated PET/MR

**DOI:** 10.1186/s13550-024-01068-8

**Published:** 2024-01-25

**Authors:** Sheng Bi, Shaozhen Yan, Zhigeng Chen, Bixiao Cui, Yi Shan, Hongwei Yang, Zhigang Qi, Zhilian Zhao, Ying Han, Jie Lu

**Affiliations:** 1 Department of Radiology & Nuclear Medicine, Xuanwu Hospital, Capital Medical University, 45 Changchun Street, Xicheng District, Beijing, 100053 China; 2 Beijing Key Laboratory of Magnetic Resonance Imaging and Brain Informatics, Beijing, China; 3 Key Laboratory of Neurodegenerative Diseases, Ministry of Education, Beijing, China; 4 Department of Neurology, Xuanwu Hospital, Capital Medical University, Beijing, China

**Keywords:** Alzheimer’s disease, Mild cognitive impairment, Arterial spin labeling MRI, ^18^F-FDG PET, Integrated PET/MR

## Abstract

**Background:**

Developing biomarkers for early stage AD patients is crucial. Glucose metabolism measured by ^18^F-FDG PET is the most common biomarker for evaluating cellular energy metabolism to diagnose AD. Arterial spin labeling (ASL) MRI can potentially provide comparable diagnostic information to ^18^F-FDG PET in patients with neurodegenerative disorders. However, the conclusions about the diagnostic performance of AD are still controversial between ^18^F-FDG PET and ASL. This study aims to compare quantitative cerebral blood flow (CBF) and glucose metabolism measured by ^18^F-FDG PET diagnostic values in patients with Alzheimer’s disease (AD) and amnestic mild cognitive impairment (aMCI) using integrated PET/MR.

**Results:**

Analyses revealed overlapping between decreased regional rCBF and ^18^F-FDG PET SUVR in patients with AD compared with NC participants in the bilateral parietotemporal regions, frontal cortex, and cingulate cortex. Compared with NC participants, patients with aMCI exclusively demonstrated lower ^18^F-FDG PET SUVR in the bilateral temporal cortex, insula cortex, and inferior frontal cortex. Comparison of the rCBF in patients with aMCI and NC participants revealed no significant difference (*P* > 0.05). The ROC analysis of rCBF in the meta-ROI could diagnose patients with AD (AUC, 0.87) but not aMCI (AUC, 0.61). The specificity of diagnosing aMCI has been improved to 75.56% when combining rCBF and ^18^F-FDG PET SUVR.

**Conclusion:**

ASL could detect similar aberrant patterns of abnormalities compared to ^18^F-FDG PET in patients with AD compared with NC participants but not in aMCI. The diagnostic efficiency of ^18^F-FDG-PET for AD and aMCI patients remained higher to ASL. Our findings support that applying ^18^F-FDG PET may be preferable for diagnosing AD and aMCI.

**Supplementary Information:**

The online version contains supplementary material available at 10.1186/s13550-024-01068-8.

## Key points

1. Hypometabolic brain areas are more widespread in patients with AD and aMCI than hypoperfused brain areas.
2. ^18^F-FDG PET provides superior diagnostic performance over ASL for patients with AD and aMCI.
3. After comprehensive consideration, PET alone is recommended for the diagnosis of AD and aMCI patients.

**Supplementary Information:**

The online version contains supplementary material available at 10.1186/s13550-024-01068-8.

## Introduction

Alzheimer’s disease (AD) is a kind of progressive neurodegenerative disorder characterized by impairments in cognitive dysfunction [1]. Currently, China has more than 10 million AD patients, making it the country with the largest number of AD patients in the world [2]. AD affects millions globally without effective treatment options, and its pathogenesis is still unclear. Amnestic mild cognitive impairment (aMCI), characterized by memory loss as its principal manifestation, represents a primary subtype of mild cognitive impairment (MCI) that exhibits a higher likelihood of transitioning to typical AD [3–5]. Approximately 16.5% of aMCI patients progress to AD annually [6]. Clinicians cannot recognize even the early indications of AD until substantial damage has occurred to essential biological components. Therefore, developing biomarkers for early stage AD patients is crucial to enable early intervention and delay or even prevent the onset of clinical symptoms.

Glucose metabolism measured by fluorine 18 (^18^F) fluorodeoxyglucose (FDG) positron emission tomography (PET) remains the most common, widely used, or well-established biomarker for evaluating cellular energy metabolism to diagnose AD, typically represented in standard uptake value ratio (SUVR) [7]. Even in the MCI stage, ^18^F-FDG PET has shown high diagnostic accuracy with AUC from 0.71 ~ 0.90, sensitivity ranging from 57 to 85% and specificity ranging from 67 to 91% [8–10]. Patients with AD also have reduced cerebral blood flow (CBF) and a state of regional hypoperfusion [11]. Due to the coupling of perfusion to glucose metabolism, arterial spin labeling (ASL) MRI, which uses endogenous arterial blood as a tracer to quantify CBF, can potentially provide comparable diagnostic information to ^18^F-FDG PET in patients with neurodegenerative disorders [12–14]. Since ASL is entirely non-invasive and free from the radiation of PET, it presents a great prospect for cost-effective monitoring of AD progression and treatment outcomes.

Previous studies have compared ^18^F-FDG PET to ASL for the diagnosis of neurodegenerative disorders [12, 13, 15–19]. However, the conclusions of diagnostic performance are still controversial. In a simultaneous PET/MR study comparing ASL and ^18^F-FDG in AD and MCI, voxel-wise analysis using pulsed ASL revealed no CBF reductions between MCI and controls, in contrast to ^18^F-FDG PET with hypometabolism in the bilateral inferior parietal cortex, posterior cingulate cortex and precuneus [18]. However, in the study of Dolui et al. [20], CBF and ^18^F-FDG-PET performed on separate PET and MR systems showed abnormalities in similar areas, particularly in medial temporoparietal regions. The variety of the small sample size, the different ASL procedures, and the inherent limits of separate PET and MRI scans may be the causes of these contradictory results [12, 15, 19]. Integrated PET/MR provides highly spatially and temporally aligned images that evaluate brain structural, functional, and metabolic information simultaneously. Therefore, this study aimed to explore the CBF and ^18^F-FDG PET SUVR in patients with AD and aMCI on integrated PET/MR basis using voxel-, region of interest (ROI)-based, and receiver operating characteristic (ROC) analyses to compare the diagnostic performance of ASL and ^18^F-FDG PET at the group level.

## Materials and methods

### Participants

A total of 137 right-handed subjects from May 10, 2018, to July 2022 were retrospectively entered into this study, comprising 47 normal control (NC) participants, 45 patients with aMCI, and 45 patients with AD. Ethical approval was obtained from the Medical Research Ethics Committee of Xuanwu Hospital, Capital Medical University. Written informed consent was obtained from each participant and/or their legal representative before the PET/MR scan. Clinical diagnosis was established based on a standard dementia screening, including medical history review, physical and neurological examinations, laboratory tests, neuropsychological tests, and brain ^18^F-FDG PET/MR scans. Patients with AD fulfilled the National Institute of Neurological and Communicative Diseases and Stroke/Alzheimer’s Disease and Related Disorders Association criteria for probable AD [21, 22]. The diagnostic criteria for aMCI, which were adapted from the MCI diagnostic criteria of Petersen, were as follows: (1) memory complaints, preferably corroborated by an informant; (2) objective memory impairment; (3) preservation of general cognitive function; (4) intact activities of daily living; and (5) absence of dementia [4, 5, 23]. All participants were assessed by a neurologist with expertise in AD disorders. Exclusion criteria were diabetes, severe white matter injury (Fazekas scores higher than 2) and other neurologic, psychiatric, or brain parenchyma diseases (e.g., stroke, tumors, and trauma) potentially leading to cognitive impairment. Two experienced neuroradiologists assessed the Fazekas scores to estimate cerebral microvascular impairment. The interval between neuropsychological assessments and simultaneous PET/MR scans was within 30 days.

### PET/MR data acquisition

Imaging data were collected with an integrated simultaneous time-of-flight (ToF) PET/MR (Signa PET/MR, GE Healthcare, WI, USA). PET and MR images were simultaneously acquired in 19-channel head and neck union coil. Each participant was given instructions to abstain from eating for a minimum of 6 h to achieve a serum glucose level below 7 mmol/L. Participants were scanned under resting conditions with their eyes closed. The PET/MR acquisition protocol was the same as in our previous studies [24–26]. MRI sequence parameters were as follows: Sagittal T1-weighted three-dimensional (3D) turbo field echo, repetition time/echo time = 8.5 ms/3.2 ms, flip angle = 15°, voxel size = 1 × 1 × 1 mm^3^, and several slices = 188. Ten-min PET and 3D pseudo-continuous ASL (pCASL) data were acquired simultaneously. ^18^F-FDG PET acquisition started 40 min after 5.6–8.2 mCi ^18^F-FDG tracer injection, with a 10-min PET images scan acquired with 3D list-mode. Detailed information on PET attenuation correction and reconstruction has been described in our previous articles [24, 26]. The reconstructed PET image matrix was 192 × 192, with a field of view of 350 × 350 mm^2^ and a voxel size of 1.82 × 1.82 × 2.78 mm^3^. The spatial resolution of the images was 4.1 mm. For 3D pCASL, the following parameters were applied: repetition time/echo time = 5362 ms/ 11.3 ms, matrix size = 64 × 64, flip angle = 111°, voxel size = 3.75 × 3.75 × 4.00 mm^3^, slice thickness = 4 mm, number of slices = 36. The post-labeling delay (PLD) was 2.0 s, consistent with previous AD or MCI studies [27, 28].

### ASL and ^18^F-FDG PET data analysis

The raw ASL data were transferred to the GE Advantage Workstation 4.7 and post-processed by the ReadyView software (version 10.3.67) in the FuncTool environment to generate whole-brain CBF maps. Both ^18^F-FDG PET and CBF data were processed using Statistical Parametric Mapping (SPM12, Wellcome Department of Imaging Neuroscience, London, United Kingdom). All the ^18^F-FDG PET and CBF images were coregistered to the individuals’ structural MRI images and normalized to standard Montreal Neurologic Institute (MNI) space using an MRI template. Then, the ^18^F-FDG PET images were transformed into maps representing the SUVR using pons as a reference region [29, 30]. The CBF images were also transformed into maps representing the relative CBF (rCBF), which were computed by normalizing the CBF in each voxel by mean CBF in putamen [20, 31]. Finally, all images were smoothed using an isotropic Gaussian kernel at full width at half maximum (FWHM) of 8 mm in all directions.

### Statistical analysis

Statistical analyses were performed using Statistical Package for Social Science version 26.0. Continuous variables with normal distribution were presented as mean ± standard deviation (SD) and compared between groups by independent T test; while categorical variables were presented as counts (with percentages) and tested using the Chi-square test. To compare the difference in ^18^F-FDG PET SUVR and rCBF images between groups, the voxel-based, and ROI-based two-sample T tests were performed using SPM12 software. Gender and age were utilized as uninteresting covariates to minimize their potential impact on cerebral metabolism and perfusion [32]. The voxel-based two-sample T tests of absolute CBF were also assessed between groups. Voxel-based group differences using 3D pCASL were also investigated by estimating rCBF with pons as the reference region, same as ^18^F-FDG PET SUVR. The defined ROIs were the same as in our previous study [33]. We also examined a customized meta-ROI constructed by using a voxel number-weighted mean of median uptakes in the coordinates of precuneus, parietal, posterior cingulate gyrus, and inferior temporal gyrus, which were significantly affected in the AD continuum [12, 17, 34, 35]. The logistic regression model was constructed utilizing SUVR and rCBF of the meta-ROI as independent variables and binary outcomes as dependent variables in the integrated analysis. Based on the model, the predictive values of logistic regression for each patient were obtained, and the ROC analysis was performed on the discriminative efficacy of the outcomes using this predictive value. The sensitivity and specificity reported in this study are generated at the best cutoff point, which is determined by the Youden index (sensitivity + specificity − 1). Uncorrected *P* < 0.001 at voxel level, Gaussian random field (GRF) corrected *P* < 0.01 at voxel level and *P* < 0.05 at cluster level were considered as of statistical significance.

## Results

### Demographics and clinical characteristics

A total of 137 subjects comprising 47 NC participants, 45 patients with aMCI, and 45 patients with AD were included in this study. The demographic characteristics of all participants are listed in Table 1. Significant group differences were found in age, MMSE, MOCA, and CDR scores (all *P* < 0.05) but not found in gender and education (*P* > 0.05). AD and aMCI groups had significantly lower MMSE, MOCA scores, and higher CDR scores than NC participants (all *P* < 0.001).

**Table 1:** Demographics of the cohort

	NC (N = 47)	aMCI (N = 45)	AD (N = 45)	*P* value (NC vs. aMCI)	*P* value (NC vs. AD)	*P* value (aMCI vs. AD)
Female (percentage)	30 (63.8%)	28 (62.2%)	27 (60.0%)	> 0.999	0.870	> 0.999
Age	61.96 (10.34)	68.56 (8.24)	64.18 (9.03)	0.001	0.276	0.018
Education years	13.82 (15.94)	11.90 (3.72)	11.15 (3.95)	0.460	0.306	0.384
MMSE	28.54 (1.62)	26.23 (3.00)	17.14 (7.50)	< 0.001	< 0.001	< 0.001
MoCA	25.89 (2.79)	21.95 (4.05)	12.79 (6.99)	< 0.001	< 0.001	< 0.001
CDR	0.04 (0.13)	0.47 (0.22)	1.19 (0.91)	< 0.001	< 0.001	< 0.001

Data were presented with mean (standard deviation). Group comparisons: independent T test (age, education, MMSE, MoCA, and CDR), gender (chi-square test). MMSE = Mini-Mental State Examination, MoCA = Montreal Cognitive Assessment, CDR = Clinical Dementia Rating. Statistical significance set at *P* < 0.05

### Voxel-wise-based group differences of ^18^F-FDG PET SUVR

**Fig. 1:**
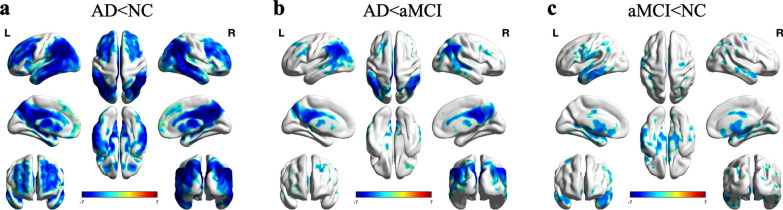
Spatial maps of the voxel-wise-based analysis with the patterns of reduced ^18^F-FDG PET SUVR with pons as the reference region in patients with AD compared with NC participants (**a**), patients with AD compared with aMCI (**b**), and patients with aMCI compared with NC participants (**c**) (voxels level with* P* < 0.01, cluster level with *P* < 0.05, GRF corrected). Colors indicate t scores

The voxel-wise-based analysis showed significantly reduced ^18^F-FDG PET SUVR in the bilateral parietotemporal regions, frontal cortex, cingulate cortex, and regions in the subcortical gray matter such as amygdala and caudate in patients with AD compared with NC participants. In patients with AD compared with patients with aMCI, a pattern of reduced ^18^F-FDG PET SUVR in the bilateral parietotemporal cortex, frontal cortex, and precuneus was found. The aMCI group displayed a pattern of significantly reduced ^18^F-FDG PET SUVR in the bilateral temporal cortex, insula cortex, fusiform gyrus, middle cingulate cortex, hippocampus, and parahippocampus compared with NC participants (*P* < 0.01, GRF corrected). Voxel-wise spatial maps of the ^18^F-FDG PET SUVR showing the respective regional patterns of reduced ^18^F-FDG uptake are presented in Fig. 1.

### Voxel-wise-based group differences of rCBF and absolute CBF

**Fig. 2:**
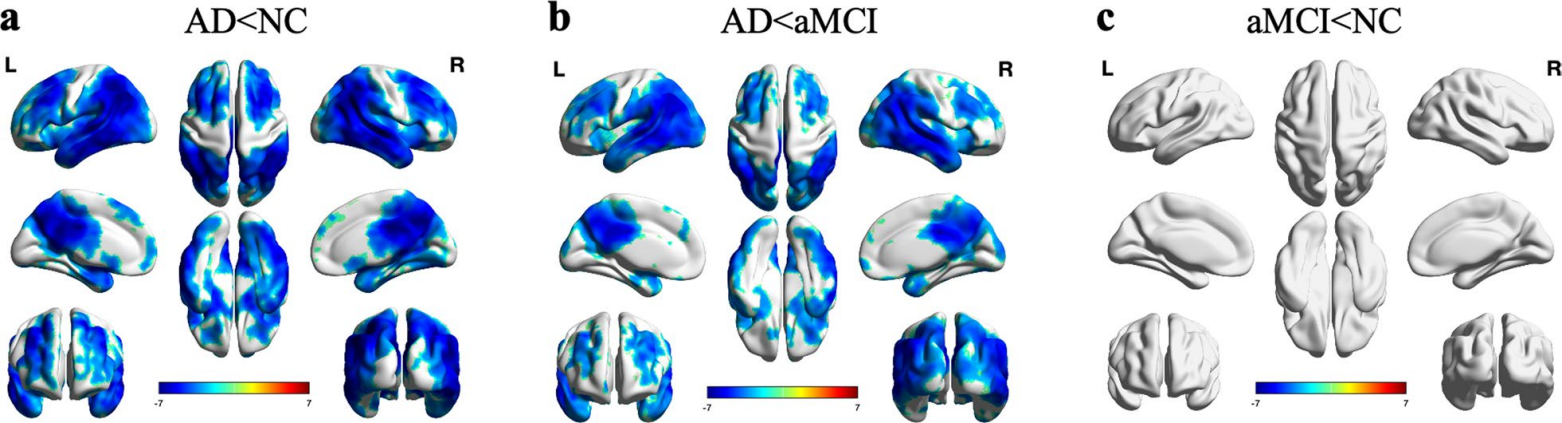
Spatial maps of the voxel-wise analysis with the patterns of reduced rCBF with putamen as the reference region in patients with AD compared with NC participants (**a**), patients with AD compared with aMCI (**b**), and patients with aMCI compared with NC participants (**c**) (voxels level with* P* < 0.01, cluster level with *P* < 0.05, GRF corrected). Colors indicate t scores

The voxel-wise analysis demonstrated a similar pattern of reduced rCBF and absolute CBF in bilateral parietotemporal regions, precuneus, frontal cortex, and posterior and middle cingulate cortex in patients with AD compared with NC participants. In patients with AD compared with aMCI, reduced rCBF and absolute CBF in bilateral parietotemporal regions, precuneus, posterior, and middle cingulate cortex were found (*P* < 0.01, GRF corrected). However, no significant rCBF and absolute CBF reductions were found in aMCI compared with NC participants (*P* > 0.05, GRF corrected). Voxel-wise spatial maps of the rCBF with putamen as the reference region showing the respective regional patterns of reduced rCBF are presented in Fig. 2. Voxel-wise spatial maps of the absolute CBF are presented in Additional file 1: Fig. S1.

To evaluate the same normalization region used for ^18^F-FDG PET SUVR, we also obtained group differences with rCBF data using pons as the reference region. In AD patients, reduced rCBF extent using pons as the reference region was larger than that with putamen as the reference region. Comparison of the rCBF in patients with aMCI and NC participants still revealed no significant difference (*P* > 0.05, GRF corrected). Voxel-wise spatial maps of the rCBF showing the respective regional patterns of reduced rCBF are presented in Additional file 1: Fig. S2.

### ROI-based group differences

The results of ROI analyses with ^18^F-FDG PET SUVR and rCBF are shown in Tables 2 and 3. In patients with AD compared with NC participants, both hypometabolic (SUVR) and hypoperfused (rCBF) regions were found in frontal, temporal, parietal lobes, hippocampus, precuneus, and cingulate cortex. Hypometabolism was observed in the medial temporal, lateral temporal, inferior temporal, hippocampus, and parahippocampus in patients with aMCI relative to NC. However, rCBF results demonstrated no significant hypoperfusion in aMCI group (*P* > 0.05).

**Table 2:** Group differences in rCBF relative to the putamen

	Relative CBF (rCBF)			*P* value		
Parameter	NC	aMCI	AD	NC vs. aMCI	NC vs. AD	aMCI vs. AD
Orbitofrontal cortex	1.18 (0.16)	1.15 (0.21)	1.06 (0.17)	0.411	0.001	0.029
Prefrontal cortex	1.27 (0.11)	1.23 (0.16)	1.16(0.14)	0.234	< 0.001	0.027
Superior frontal cortex	1.19 (0.10)	1.17 (0.11)	1.05 (0.13)	0.371	< 0.001	< 0.001
Lateral temporal	1.35 (0.15)	1.31 (0.17)	1.11 (0.18)	0.283	< 0.001	< 0.001
Medial temporal	1.02 (0.12)	0.99 (0.10)	0.91 (0.11)	0.193	< 0.001	0.001
Inferior temporal	1.24 (0.15)	1.20 (0.14)	1.03 (0.18)	0.258	< 0.001	< 0.001
Parietal	1.32 (0.16)	1.27 (0.19)	1.02 (0.25)	0.166	< 0.001	< 0.001
Posterior precuneus	1.51 (0.17)	1.44 (0.21)	1.17 (0.28)	0.089	< 0.001	< 0.001
Posterior cingulate	1.65 (0.20)	1.59 (0.21)	1.27 (0.27)	0.134	< 0.001	< 0.001
Hippocampus	1.10 (0.12)	1.07 (0.11)	0.99 (0.14)	0.253	< 0.001	0.003
Parahippocampus	1.06 (0.13)	1.01 (0.12)	0.95(0.13)	0.053	< 0.001	0.036

Data were presented with mean (standard deviation). There was a significantly decreased rCBF in AD compared to NC, and AD compared to aMCI. There were no significant differences between aMCI and NC in rCBF (*P* value ranged from 0.053 to 0.898). *P* < 0.05 indicate statistical significance

**Table 3:** Group differences in ^18^F-FDG PET SUVR relative to the pons

Parameter	^18^F-FDG SUVR			*P* value		
	NC	aMCI	AD	NC vs. aMCI	NC vs. AD	aMCI vs. AD
Orbitofrontal cortex	0.61 (0.06)	0.60 (0.09)	0.57 (0.07)	0.595	0.005	0.064
Prefrontal cortex	0.68 (0.06)	0.65 (0.10)	0.62 (0.08)	0.108	< 0.001	0.039
Superior frontal cortex	0.73 (0.06)	0.71 (0.09)	0.63 (0.10)	0.323	< 0.001	< 0.001
Lateral temporal	0.63 (0.05)	0.59 (0.07)	0.53 (0.07)	0.001	< 0.001	0.001
Medial temporal	0.50 (0.04)	0.47 (0.07)	0.46 (0.05)	0.001	< 0.001	0.917
Inferior temporal	0.61 (0.05)	0.58 (0.05)	0.53 (0.07)	0.003	< 0.001	< 0.001
Parietal	0.67 (0.06)	0.65 (0.10)	0.51 (0.10)	0.155	< 0.001	< 0.001
Posterior precuneus	0.82 (0.07)	0.81 (0.11)	0.62 (0.13)	0.413	< 0.001	< 0.001
Posterior cingulate	0.84 (0.07)	0.82 (0.12)	0.63 (0.11)	0.358	< 0.001	< 0.001
Hippocampus	0.52 (0.04)	0.48 (0.06)	0.45 (0.06)	< 0.001	< 0.001	0.039
Parahippocampus	0.52 (0.05)	0.48 (0.06)	0.46 (0.05)	0.001	< 0.001	0.081

Data were presented with mean (standard deviation). There was a significantly decreased ^18^F-FDG PET SUVR in AD compared to NC in all 11 ROIs, AD compared to aMCI in 8 ROIs, and aMCI compared to NC in 5 ROIs. *P* < 0.05 indicate statistical significance

### ROC analysis for disease classification

**Fig. 3:**
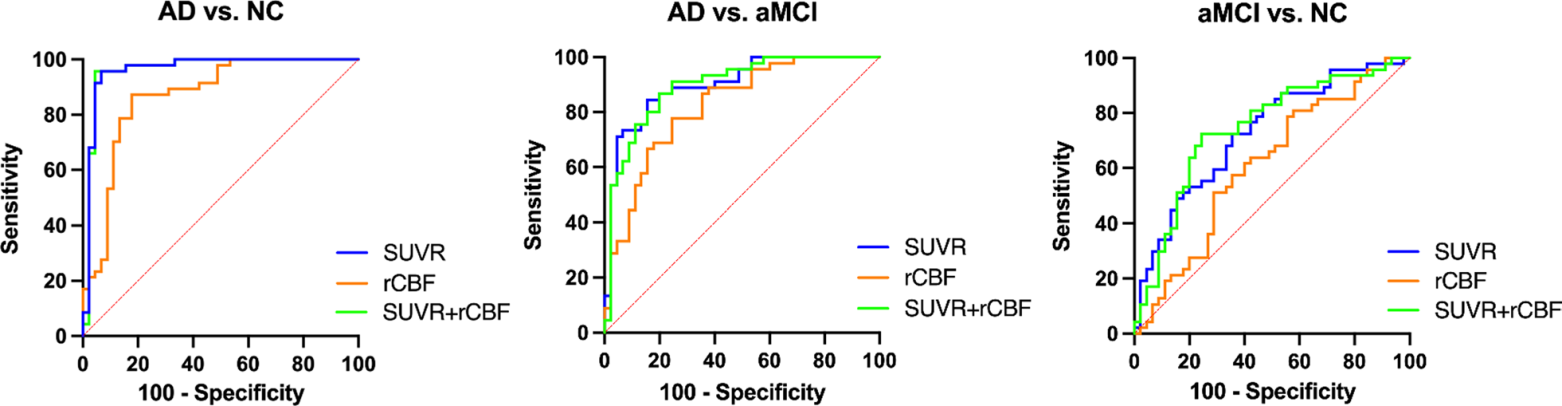
ROC curves in differentiating patients with AD from NC participants, AD from aMCI, and aMCI from NC using ^18^F-FDG PET SUVR (blue), rCBF (orange), and ^18^F-^18^F-FDG PET SUVR combined with rCBF (green). SUVR, standard uptake value ratio. rCBF, relative cerebral blood flow. SUVR + rCBF, standard uptake value ratio combines with relative cerebral blood flow

For performance comparison, AUC was calculated for ^18^F-FDG SUVR and rCBF. Using the meta-ROI, ^18^F-FDG SUVR and rCBF had AUC (sensitivity, specificity) of 0.96 (95.74%, 93.30%) and 0.87 (87.23%, 82.22%) in differentiating patients with AD and NC. In predicting patients with aMCI, all methods demonstrated moderate discriminatory power. ^18^F-FDG SUVR and rCBF had AUC of 0.73 (72.34%, 64.44%), 0.61 (78.72%, 44.44%). In differentiating patients with AD from aMCI, ^18^F-FDG SUVR and rCBF had AUC of 0.90 (84.44%, 84.44%), 0.82 (77.78%, 75.56%). After combining ^18^F-FDG PET and rCBF, the specificity of diagnosing aMCI has been improved to 75.56%. The ROC curve results are shown in Table 4 and Fig. 3.

**Table 4:** ROC curves results of meta-ROI in identification for AD, aMCI and NC

	AUC			Sensitivity(%)			Specificity(%)		
	SUVR	rCBF	SUVR + rCBF	SUVR	rCBF	SUVR + rCBF	SUVR	rCBF	SUVR + rCBF
NC vs. aMCI	0.728	0.610	0.744	72.34	78.72	72.34	64.44	44.44	75.56
NC vs. AD	0.963	0.869	0.963	95.74	87.23	95.74	93.30	82.22	95.56
aMCI vs. AD	0.901	0.824	0.897	84.44	77.78	86.67	84.44	75.56	80.00

SUVR was calculated from ^18^F-FDG PET images. AUC, area under ROC curve. SUVR, standard uptake value ratio. rCBF, relative cerebral blood flow. SUVR + rCBF, standard uptake value ratio combines with relative cerebral blood flow

## Discussion

In this study, we applied integrated PET/MR based on whole-brain ^18^F-FDG PET and ASL with voxel-wise-based, ROI-based, and ROC analysis to investigate the characteristic alterations and diagnostic performance of glucose metabolism and perfusion in AD and aMCI patients. The main finding of this study was that AD patients exhibited comparable spatial abnormalities in cerebral glucose metabolism and perfusion to NC individuals. However, ^18^F-FDG PET AUC in differentiating both AD and aMCI patients from NC individuals was higher than ASL, especially in aMCI patients.

Prior research on AD has consistently identified glucose hypometabolism in the bilateral temporal–parietal, precuneus, and posterior cingulate cortex regions using both quantitative and qualitative ^18^F-FDG PET studies [13, 14, 17–19]. Similarly, studies on ASL have detected hypoperfusion in the same cerebral regions [27, 36–39], which have been linked to neuropsychological impairments and are well-established AD-related regions [40]. Consistent with prior research, the current findings of ^18^F-FDG PET and ASL demonstrate that AD is characterized by profoundly aberrant metabolism and perfusion, particularly in the posterior cingulate cortex and bilateral precuneus.

Regarding patients with MCI, ^18^F-FDG PET metabolic impairment has been reported in the parietotemporal cortex, posterior cingulate cortex, and precuneus [20, 34, 41]. The present ^18^F-FDG results are partially in line with these existing reports, as hypometabolism was observed mainly in the bilateral temporal cortex, as well as the insula cortex, hippocampus, parahippocampus, fusiform gyrus, and middle cingulate cortex in patients with aMCI compared with NC. However, neither absolute nor rCBF reduced regions were observed in the aMCI group, suggesting that hypoperfusion becomes more pronounced as AD progresses [42]. Since Aβ plaques and neurofibrillary tangles are typical pathological features of AD, the neurotoxic effects of Aβ can impair vascular function and cause cerebral hypoperfusion, jointly promoting cognitive dysfunction. Furthermore, an inverse correlation was observed between CBF and tau in the temporoparietal cortex. However, it was observed that patients with a greater burden of Aβ exhibited a stronger CBF-tau relationship. As a result, a greater comprehension of the pertinent pathophysiological mechanisms can be attained through the investigation of cerebral perfusion in patients with AD and aMCI [43–45]. However, to date, the outcomes of ASL research involving patients with MCI have been inconclusive. Pseudo-continuous ASL studies found reduced CBF in the bilateral parietal, precuneus, medial temporoparietal cortex, posterior cingulate gyrus, and subcortical gray matter nuclei in MCI patients compared to NC [20, 27]. However, Riederer et al. [18] applying pulsed ASL did not find significantly hypoperfused cerebral regions in MCI patients. These studies only focused on patients with MCI. Among them, aMCI has a higher propensity for progressing to typical AD, whereas non-aMCI is more likely to develop into other forms of dementia, including vascular dementia and Lewy body dementia. Hence, investigating alterations in cerebral glucose metabolism and blood flow during aMCI could potentially yield more valuable insights into the pathophysiological mechanisms underlying early AD and provide an imaging basis for early detection. The results of this study supported the hypothesis that ^18^F-FDG PET is superior to ASL-MRI for the diagnosis of patients in the early clinical stage of AD, suggesting that the alteration of brain glucose metabolism in the early clinical stage of AD may occur before CBF changes and other abnormalities [46].

In contrast, this study focuses on aMCI patients who may proceed to AD, and still no reduction in rCBF or absolute CBF brain region was found. Since the severity of white matter hyperintensity in AD patients may be related to CBF in some brain regions, only patients with Fazekas scores smaller than 2 were included in this study to avoid the possible influence of white matter hyperintensity on CBF [27]. However, establishing a consensus on the clinical utility of ASL in the diagnosis of AD or MCI from prior studies is limited by heterogeneity in ASL techniques, diversity of study populations such as mixed dementia subtypes, use of relatively small sample sizes, heterogeneity of cohorts, differences in ASL techniques and image analysis methodology. For instance, the difference in ASL techniques (label scheme, PLD) can significantly impact the signal-to-noise ratio (SNR), leading to image artifacts such as motion and altering perceived contrast (grey-to-white matter ratio) [47]. Our choice of PLD was implemented in the ASL whitepaper, which recommended 2.0 s for imaging older brains. Because ASL is inherently sensitive to motion and susceptibility distortion artifacts, which further minimize the already low SNR and significantly impact sensitivity, future studies will need to improve using higher-phased array receiver coils such as 32/64 channel head coils.

The selection of the pons as the reference location for ^18^F‐FDG SUVR in this investigation was based on previous research that has shown its maintained metabolism in individuals with Alzheimer’s disease. As a result, the pons can be considered a reliable mechanism for normalizing data. Furthermore, research findings by Li et al. revealed that the reference tissue pons‐based ^18^F‐FDG SUVR exhibits superior sensitivity in identifying longitudinal alterations compared with the reference tissue cerebellum- and centrum semiovale-based ^18^F‐FDG SUVR [29, 48–50]. The putamen was selected as the reference region for rCBF in this study because it does not exhibit hypoperfusion in the early stages of AD and is non-differential across groups in the voxel-wise-based absolute CBF analysis of this study [20, 31]. In contrast, the cerebellum was not selected as a reference brain region for rCBF since it is more susceptible to artifacts due to its inferior location.

In addition, we performed a partial volume correction (PVC) using the Muller–Gartner approach implemented in SPM toolbox PET-PVE12 on the ^18^F-FDG PET images to avoid partial volume effects due to atrophy. The results showed that the results after PVC were roughly similar to the voxel-wise results without correction, consistent with some previous research results [20, 27, 51]. But the hypometabolism cerebral regions after PVC in the aMCI group were relatively more extensive, mainly located in the parietal and insular cortex. After PVC, the specificity increased from 64.44 to 84.44. However, the AUC values of both were similar (with PVC: 0.731 vs. without PVC: 0.728). Voxel-wise spatial maps of reduced ^18^F-FDG PET SUVR with and without PVC are presented in Additional file 1: Fig. S3. The results of ROI analyses and ROC curves for ^18^F-FDG PET SUVR with PVC are shown in Additional file 1: Tables S1 and S2.

Our qualitative findings were corroborated by the results of our quantitative assessments utilizing meta-ROI analysis at the group level in hypoperfusion and hypometabolic regions. We assessed the diagnostic effectiveness of ^18^F-FDG PET and ASL by utilizing a meta-ROI comprising the inferior temporal gyrus, precuneus, parietal, and posterior cingulate gyrus, which are the brain regions most commonly impacted in AD patients [12, 17, 20, 34, 35]. In contrast to ASL (AUC: 0.87), ^18^F-FDG PET (AUC: 0.96) demonstrated superior performance in distinguishing patients with AD from NC participants, according to the findings of the present study. However, only a few studies have evaluated the diagnostic efficacy with a largely varies among studies of AUC from 0.71 ~ 0.90 for ^18^F-FDG PET and 0.74 ~ 0.90 for ASL in differentiating patients with MCI from NC participants [10, 19, 20]. Our findings indicate that ^18^F-FDG PET has a greater specificity in distinguishing patients with aMCI from NC whereas ASL demonstrates a higher sensitivity. With the combination of ^18^F-FDG PET and rCBF, the specificity of the diagnosis of aMCI was raised to 75.56%, illustrating the potential of PET/MR for the accurate diagnosis of MCI. But the overall AUC is comparable to that of ^18^F-FDG PET alone. Given the fact that ^18^F-FDG FDG PET voxel analysis has demonstrated a greater capacity to identify abnormally hypometabolic brain regions compared to ASL, along with the restricted accessibility and supplementary expenses associated with combined PET/MR, our recommendation is to only employ ^18^F-FDG PET in clinical environments for the diagnosis of aMCI.

A potential limitation of this study is that the recruitment of participants was not guided by the use of CSF and/or PET Aβ and tau biomarkers. Additionally, the aMCI group included in this study was older than the NC group, which could have affected the brain metabolism and perfusion patterns of the aMCI patients [52]. Another limitation was that compared to ^18^F-FDG PET, the effects of the SNR and artifacts made the cerebral perfusion measured by ASL variable across individuals [53]. A head coil with more receiver channels, as opposed to the 19-channel coil used in this study, may improve the SNR of the images as well as the sensitivity of detecting abnormal alteration patterns. Finally, due to the lack of longitudinal data, the impact of our findings on the transition from NC to aMCI and AD remains unidentified, and future follow-up studies are required.

## Conclusions

To conclude, ASL could detect similar aberrant patterns of abnormalities compared to ^18^F-FDG PET using integrated PET/MR data in patients with AD compared with NC participants, but not in aMCI. The diagnostic efficiency of ^18^F-FDG-PET for AD and aMCI patients remained higher to ASL. Therefore, applying ^18^F-FDG PET may be preferable for diagnosing AD and aMCI.

### Supplementary Information

**Additional file 1:** Absolute CBF, rCBF with pons as reference, and F-FDG PET SUVR with PVC data analysis results.

## Data Availability

The datasets generated during the current study are available from the corresponding author on reasonable request.

## Abbreviations

AD: Alzheimer’s disease
aMCI: Amnestic mild cognitive impairment
SUVR: Standard uptake value ratio
ASL: Arterial spin labeling
rCBF: Relative cerebral blood flow
AUC: Area under ROC curve
