# Supplementary material for: Comparison of 18F-FDG PET and arterial spin labeling MRI in evaluating Alzheimer’s disease and amnestic mild cognitive impairment using integrated PET/MR
Source: EJNMMI Res. 2024 Jan 25;14:9. doi: 10.1186/s13550-024-01068-8 (PMC10811308; doi:10.1186/s13550-024-01068-8)
Supplement: Supplementary file 1 — Additional file 1: Absolute CBF, rCBF with pons as reference, and F-FDG PET SUVR with PVC data analysis results. [file 13550_2024_1068_MOESM1_ESM.docx]

**Supplementary material**

**
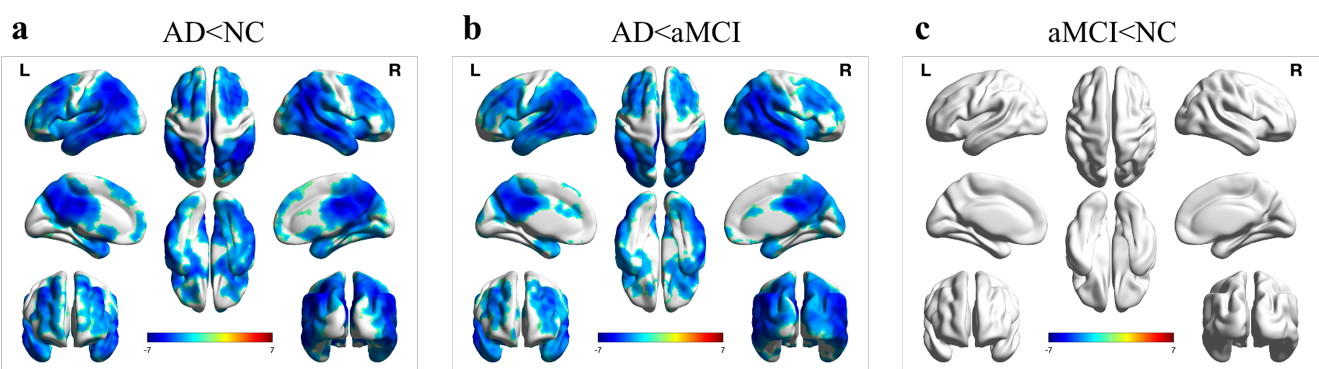
**

Fig. 1 Spatial maps of the voxel-wise analysis with the patterns of absolute CBF in patients with AD compared with NC participants (a), patients with AD compared with aMCI (b), and patients with aMCI compared with NC participants (c) (voxels level with P < 0.01, cluster level with P < 0.05, GRF corrected). Colors indicate t scores.


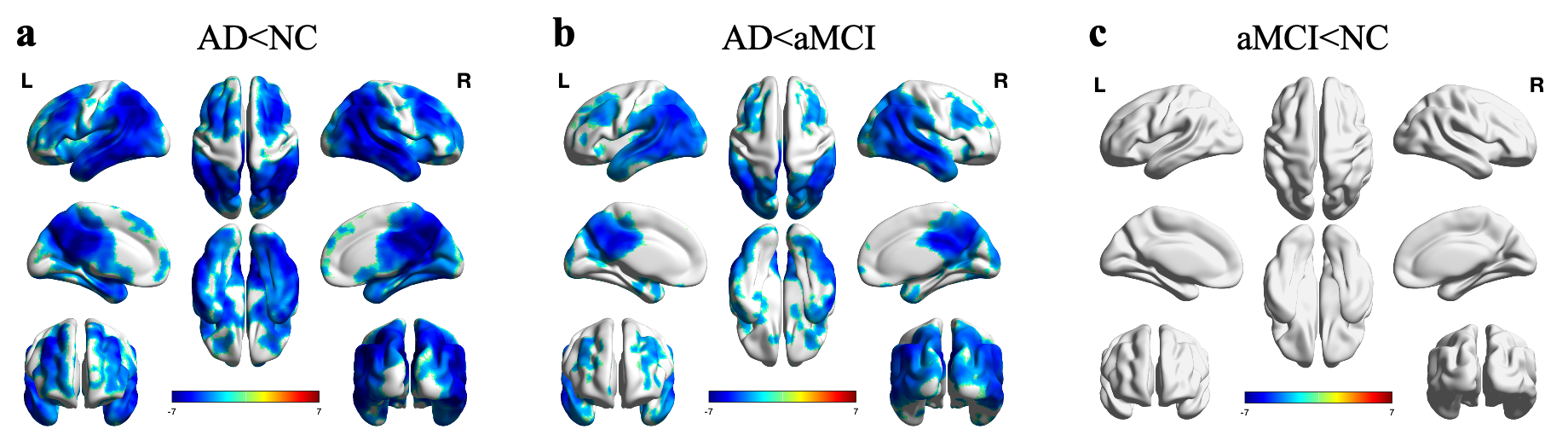


Fig. 2 Spatial maps of the voxel-wise analysis with the patterns of reduced rCBF with pons as the reference region in patients with AD compared with NC participants (a), patients with AD compared with aMCI (b), and patients with aMCI compared with NC participants (c) (voxels level with *P* < 0.01, cluster level with *P* < 0.05, GRF corrected). Colors indicate t scores.


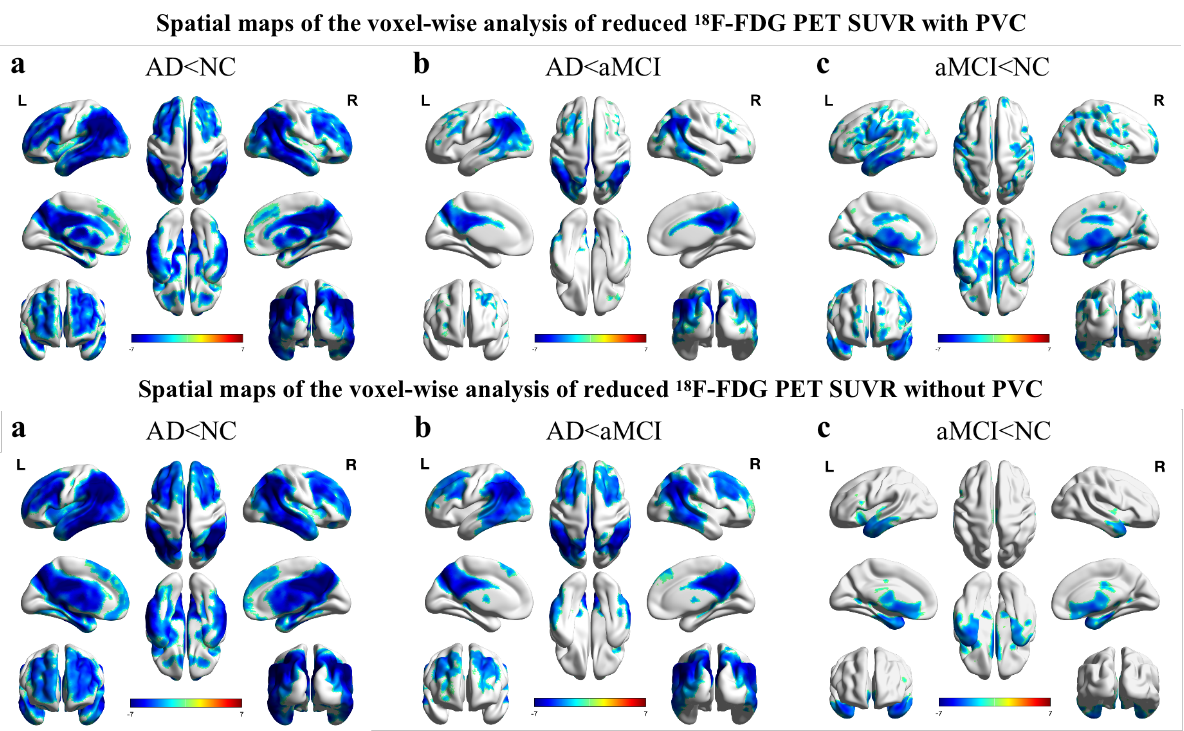


Fig. 3 Spatial maps of the voxel-wise analysis with the patterns of reduced ^18^F-FDG PET SUVR with (row 1) and without PVC (row 2) in patients with AD compared with NC participants (a), AD compared with aMCI (b), and aMCI compared with NC (c) (voxels level with *P* < 0.01, cluster level with *P* < 0.05, GRF corrected). Colors indicate t scores. Compared with voxel-wise analysis without PVC, patients with aMCI after PVC showed a wider range of hypometabolic brain regions, mainly concentrated in the parietal and insular cortex.

**Table1 ROC curves results of ^18^F-FDG PET SUVR of meta-ROI in identification for AD, aMCI, and NC with and without PVC**

|  | AUC | |  | Sensitivity（%） | |  | Specificity（%） | |
| --- | --- | --- | --- | --- | --- | --- | --- | --- |
|  | with PVC | without PVC |  | with PVC | without PVC |  | with PVC | without PVC |
| NC vs. aMCI | 0.731 | 0.728 |  | 55.32 | 72.34 |  | 84.44 | 64.44 |
| NC vs. AD | 0.939 | 0.963 |  | 93.62 | 95.74 |  | 91.11 | 93.30 |
| aMCI vs. AD | 0.863 | 0.901 |  | 80.00 | 84.44 |  | 91.11 | 84.44 |

AUC area under ROC curve

PVC partial volume correction

SUVR was calculated relative to the pons. After PVC correction, the sensitivity for identifying aMCI and NC decreased from 72.34 to 55.32, but the specificity increased from 64.44 to 84.44 for identifying aMCI and NC and from 84.44 to 91.11 for identifying aMCI and AD. However, the AUC did not change significantly.

**Table 2 Group differences in ^18^F-FDG PET SUVR relative to the pons with PVC**

|  | ^18^F-FDG SUVR | | |  | *P* value | | |
| --- | --- | --- | --- | --- | --- | --- | --- |
| Parameter | NC | aMCI | AD |  | NC vs. aMCI | NC vs. AD | aMCI vs. AD |
| Orbitofrontal cortex | 1.02 (0.14) | 0.98 (0.20) | 0.93 (0.19) |  | 0.304 | 0.012 | 0.226 |
| Prefrontal cortex | 1.07 (0.16) | 0.99 (0.21) | 0.94 (0.17) |  | 0.046 | <0.001 | 0.184 |
| Superior frontal cortex | 1.11 (0.18) | 1.03 (0.20) | 0.92 (0.20) |  | 0.041 | <0.001 | 0.009 |
| Lateral temporal | 1.04 (0.15) | 0.89 (0.18) | 0.84 (0.15) |  | <0.001 | <0.001 | 0.141 |
| Medial temporal | 0.88 (0.11) | 0.79 (0.18) | 0.84 (0.17) |  | 0.008 | 0.236 | 0.186 |
| Inferior temporal | 1.01 (0.13) | 0.93 (0.16) | 0.84 (0.15) |  | 0.008 | <0.001 | 0.007 |
| Parietal | 1.02 (0.12) | 0.92 (0.18) | 0.68 (0.22) |  | 0.003 | <0.001 | <0.001 |
| Posterior precuneus | 1.33 (0.18) | 1.26 (0.27) | 0.96 (0.28) |  | 0.107 | <0.001 | <0.001 |
| Posterior cingulate | 1.45 (0.18) | 1.37 (0.25) | 1.04 (0.23) |  | 0.122 | <0.001 | <0.001 |
| Hippocampus | 0.96 (0.14) | 0.82 (0.19) | 0.82 (0.27) |  | <0.001 | 0.001 | 0.874 |
| Parahippocampus | 0.91 (0.14) | 0.80 (0.20) | 0.75 (0.16) |  | 0.003 | <0.001 | 0.125 |

Notes: Table 2 shows the results of ROI analysis of ^18^F-FDG PET SUVR after PVC. Data were presented with mean (standard deviation). There was a significantly decreased ^18^F-FDG PET SUVR in AD compared to NC in 10 ROIs, AD compared to aMCI in 5 ROIs, and aMCI compared to NC in 8 ROIs. *P* < 0.05 indicate statistical significance.
